# Kyste épidermoïde du troisième ventricule: une localisation rare

**DOI:** 10.11604/pamj.2014.18.120.4342

**Published:** 2014-06-06

**Authors:** Sidi Salem-Memou, Carmine Mottolese

**Affiliations:** 1 Service de Neurochirurgie B, Hôpital Neurologique et Neurochirurgical Pierre Wertheimer, Lyon, France

**Keywords:** kyste epidermoide, endoscopie, troisième ventricule, squamous cyst, endoscopy, third ventricle

## Image en medicine

Les kystes épidermoïdes ou tumeurs perlées de Cruveilhier sont des tumeurs bénignes, congénitales, développées à partir des inclusions ectodermiques. Ils se localisent classiquement au niveau de l'angle pontocérébelleux et de la région sellaire. Les localisations intraventriculaires sont rares et concernent le plus souvent le quatrième ventricule. Les kystes épidermoïdes du troisième ventricule sont exceptionnels. Nous rapportons le cas d'un homme de 49 ans, sans antécédents pathologiques notables, ayant consulté pour céphalées chroniques, baisse de la libido et troubles de la mémoire portant sur les faits récents. L'examen neurologique était normal. L'IRM cérébrale mettait en évidence une dilatation des ventricules latéraux associée à un élargissement de la cavité du troisième ventricule par une lésion kystique en hyposignal en T1 (A), en hypersignal en T2 (B), et sans rehaussement après injection de produit de contraste (A). Un abord endoscopique était réalisé. Dans la lumière du ventricule latéral, on repère le foramen interventriculaire droit avec le pilier antérieur du fornix en avant (C, flèche noire) et le plexus choroïde en arrière (C, flèche bleue). Le troisième ventricule était rempli à rebord de matériel épidermoïde blanc nacre, très visqueux (C, flèche rouge) dont l'exérèse était obtenue au moyen d'un dissecteur à ultrasons. Les suites opératoires étaient simples. L'histologie a confirmé le diagnostic de kyste épidermoïde.

**Figure 1:**
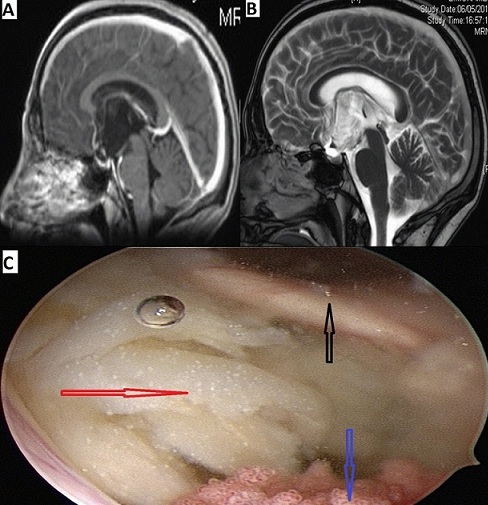
A): IRM cérébrale en coupe sagittale T1 objectivant une formation kystique du troisième ventricule en hyposignal, sans prise de contraste; B): IRM cérébrale en coupe sagittale T2 montrant le kyste du troisième ventricule en hypersignal; C): vue endoscopique montrant le foramen interventriculaire de Monroe droit, limité par le pilier antérieur du fornix en avant (flèche noire) et le plexus choroïde en arrière (flèche bleue), et la substance épidermoïde blanche nacrée remplissant a rebord le troisième ventricule (flèche rouge)

